# Quantifying Colocalization: Thresholding, Void Voxels and the *H_coef_*

**DOI:** 10.1371/journal.pone.0111983

**Published:** 2014-11-06

**Authors:** Jeremy Adler, Ingela Parmryd

**Affiliations:** 1 Department of Immunology, Genetics and Pathology, Science for Life Laboratory, Uppsala University, Uppsala, Sweden; 2 Department of Medical Cell Biology, Science for Life Laboratory, Uppsala University, Uppsala, Sweden; J. Heyrovsky Institute of Physical Chemistry, Czech Republic

## Abstract

A critical step in the analysis of images is identifying the area of interest e.g. nuclei. When the nuclei are brighter than the remainder of the image an intensity can be chosen to identify the nuclei. Intensity thresholding is complicated by variations in the intensity of individual nuclei and their intensity relative to their surroundings. To compensate thresholds can be based on local rather than global intensities. By testing local thresholding methods we found that the local mean performed poorly while the Phansalkar method and a new method based on identifying the local background were superior. A new colocalization coefficient, the *H_coef_*, highlights a number of controversial issues. (i) Are molecular interactions measurable (ii) whether to include voxels without fluorophores in calculations, and (iii) the meaning of negative correlations. Negative correlations can arise biologically (a) because the two fluorophores are in different places or (b) when high intensities of one fluorophore coincide with low intensities of a second. The cases are distinct and we argue that it is only relevant to measure correlation using pixels that contain both fluorophores and, when the fluorophores are in different places, to just report the lack of co-occurrence and omit these uninformative negative correlation. The *H_coef_* could report molecular interactions in a homogenous medium. But biology is not homogenous and distributions also reflect physico-chemical properties, targeted delivery and retention. The *H_coef_* actually measures a mix of correlation and co-occurrence, which makes its interpretation problematic and in the absence of a convincing demonstration we advise caution, favouring separate measurements of correlation and of co-occurrence.

## Introduction

Image analysis usually includes a segmentation step to differentiate between areas considered to be of interest, referred to as the foreground, and the background [1]. This is often achieved by intensity thresholding. The level at which a threshold is set is important since which pixels are designated as foreground affects subsequent quantitation. Manually set thresholds are problematic, as individuals use different or even inconsistent criteria and there is a risk of operator bias. This creates a strong case for automated thresholding, but since images vary there are many methods for setting global thresholds, ImageJ offers16 options. Complications are caused by uneven staining, background or illumination and globally set thresholds can leave parts of an image incorrectly segmented. A solution is to generate thresholds locally. An example is the local mean threshold (LMT), the arithmetic mean of the intensities of the voxels within a chosen radius, which roughly divides the pixels into two similarly sized populations and accommodates intensity gradients. However we were concerned because the LMT will still inappropriately divide the pixels into two similarly sized populations even when the local area is predominantly foreground or background.

Colocalization can be considered to cover two distinct properties, co-occurrence which reports whether the fluorophores are found together and correlation which reports on whether the intensities of the two fluorophores have similar spatial distributions [2]–[4]. Pairs of molecules can show complete co-occurrence but nonetheless be uncorrelated. This has implications for which pixels should be used in correlation measurements.

Arguably there are already too many colocalization coefficients but the newly minted *H_coef_* promises to provide an additional interpretation [5], one reflecting the interaction between molecules. It is similar to the Manders *M1* and *M2* coefficients [6] in that all pixels are used in its calculation and has similarities with the numerator of the Manders Overlap Coefficient (*MOC*). We have compared the *H_coef_* with the Pearson correlation coefficient (*r*) and the *MOC*.

A novel feature of the *H_coef_* is that empty pixels carry weight, in that a higher value is returned as the proportion of empty pixels increases and the fluorophores become concentrated in the remainder. 
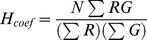
(1)


*R* and *G* are the concentrations of the two fluorophores in the same pixel, and *N* is the number of pixels.

A reasonable expectation for any new colocalization coefficient is a demonstration that it can produce useful information about changes in distribution and that it is superior to the existing coefficients. Accordingly we have examined the *H_coef_*, and related colocalization issues, considering the following points:

1. What empty pixels signify and whether they and pixels with only one type of fluorophore should be included in correlation measurements.
2. How to interpret negative correlations or values of the *H_coef_* below 1.
3. How the *H_coef_* performs with a controlled series of simulations.
4. Whether molecular interactions are measurable in living cells by colocalization analysis.

## Results

### A comparison of locally calculated thresholding methods

**Figure 1:**
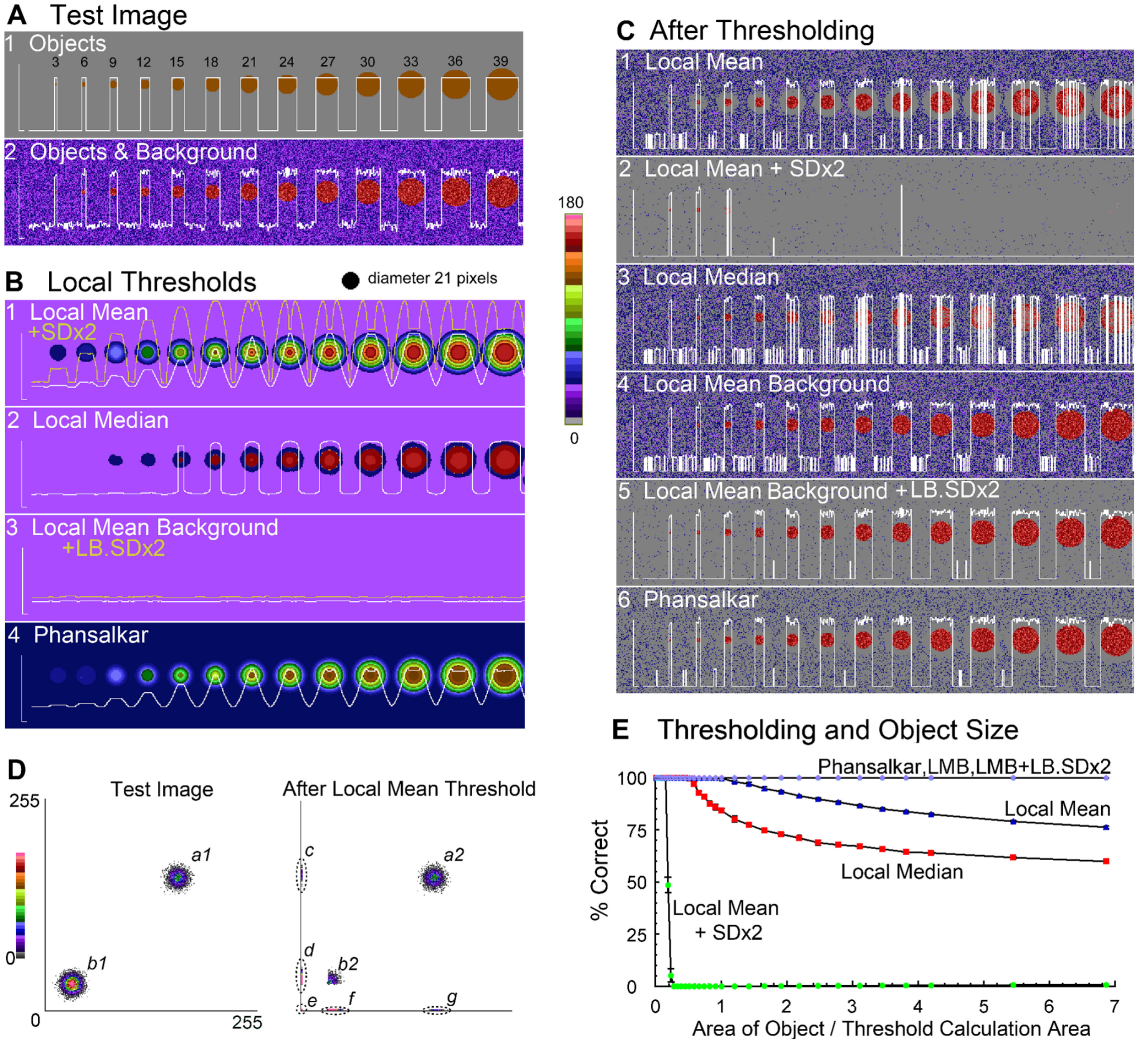
Comparison of local thresholding methods. In A, B and C each panel shows an image plus a graph (white) displaying the intensities along a horizontal line passing through the centre of the objects. The scale is 0–160. (A) Illustrates the creation of the test image from a sequence of circular objects (their diameters in pixels are superimposed) (A1) plus background with an offset and Poisson noise (A2). (B) Thresholds created from the test image using different operations over a calculation area with a diameter of 21 pixels. Note that (B1) and (B3) include a second graph (yellow), showing the primary threshold plus two standard deviations. (C) The consequences of applying six different thresholds to the test image (A2). Intensities above the threshold were retained while those below the threshold were set to zero. (D) Scattergrams showing the effect of the LMT. The source images are 62 pixel wide horizontal strips taken from the centre of the test image (A1), with noise added independently. In the scattergram showing the effects of the LMT dashed ellipses highlight new combination of intensities. (E) The size of objects and the effectiveness of different thresholding methods. Size of objects is expressed as their area as a fraction of the calculation area (diameter of 21 pixels) and effectiveness by the fraction of the area of each object that was correctly thresholded. Intensity and noise as in the test image (A2). Data shown are means +SD, N = 8. Additional analysis is shown in Table 1.

Thresholding is a basic image analysis step. When the background is inhomogenous, methods based on local rather than global intensity are preferable. To assess their performance several locally derived thresholds were applied to a simple test image, one designed to be easy to threshold, with a clear separation between the intensity range of the foreground and background and a modest amount of noise (Figure 1). A local threshold for every pixel was calculated using all the adjacent pixels within a diameter of 21 pixels. The thresholds and the consequences of setting intensities below the threshold to zero are shown in Figure 1 and Table 1. In addition the values of individual pixels along a horizontal line that runs through the midline of the objects (Figure 1A, B, C) are shown. How the size of the round foreground objects affects the different methods is shown in Figure 1E.

**Table 1:** The performance of locally set thresholds.

	Foreground (objects)				Background	
Threshold	All	Annulus	Centre	All	Annulus	Remainder
LM	87	97	47	61	86	54
LM+SDx2	2	1	2	99	100	98
Median	70	76	47	56	62	54
LMB	100	100	100	54	54	54
LMB+LB.SDx2	100	100	100	97	97	97
Phansalkar	100	100	100	85	93	82

**Figure 2:**
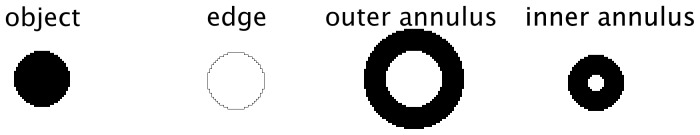
Division of the test image into regions. The analysis of the performance of locally set thresholds (Table 1) considers different parts of the image based on proximity to the edge of the objects. The object edge and the extent of the inner and outer annuli are illustrated.

The percentage of the pixels a test image that were correctly designated was measured. The test image (to Figure 1:2) was an extended version of Figure 1A2 with a wider range of diameters (1–55 pixels). Six areas were examined: the objects (foreground), the annulus inside the objects (Figure 2), their centres (objects excluding the annulus), total background, the annulus outside the objects (Figure 2) and the remaining background (excluding the outer annulus). The annulus included all pixels, outside or inside, within 10.5 pixels of the objects‘ edge. Data shown are means, N = 8.

The LMT (Figure 1B1) uses a smoothed version of the original image as the local threshold. The threshold becomes equal to the mean background or the mean foreground when the calculation radius contains only background or foreground pixels, with intermediate values appearing when mixtures of foreground and background are found within the calculation radius. Thresholding with the LMT (Figure 1C1) is unsatisfactory both in areas dominated by background where only 54% of the pixels were correctly classified and in exclusively foreground areas, at the centre of larger objects, where only 47% of the pixels were correctly designated. In areas comprising background and foreground the segmentation is effective and an outer annulus of correctly designated background pixels is apparent around each object, with 86% correctly designated, compared to only 54% in the background further from the objects. Overall to work as a threshold the LMT requires that the chosen calculation radius must exceed the size of all foreground objects and that the calculation area is not exclusively or predominantly either foreground or background. Overall this favours large calculation radii but larger radii are less able to accommodate local variations in the intensities.

The difficulty the LMT has with areas dominated by background pixels can be ameliorated by increasing the threshold to exclude almost all the background by adding twice the local standard deviation, calculated over the same area as the local mean, to the local mean (Figure 1B1, yellow graph). The LMT+SDx2 (Figure 1C2) correctly thresholds almost all the background pixels. However the success with the background is undermined by the failure to correctly designate the pixels in all but the smallest objects (Figure 1E) with a dramatic cut off at around one sixth of the area of the calculation radius.

A variant for thresholding an image with a range of foreground and background intensities is to use the local median (Figure 1B2) instead of the local mean (Figure 1B1). The local median (Figure 1B2), follows the local intensity variations in the image better than the local mean, but the final result is actually worse (Figure 1C3), because the edge enhancement seen with the LMT in the outer and inner annuli is greatly reduced, dropping from 97% to 76% and 86% to 62% respectively (Table 1).

We thought that an image segmentation method based on the mean of the local background, not just the local mean of all pixels, would circumvent the shortcomings of the LMT. We therefore tested the local mean background (LMB) (Figure 1B3), the mean of the background pixels, which resulted in a perfect designation of the objects (Figure 1C4), but like the LM the LMB unsurprisingly incorrectly designated 46% of the background pixels (Figure 1C4). This was remedied by adding twice the standard deviation of the pixels from the local background (LB.SDx2) (Figure 1B3, yellow graph), with 97% of the background pixels correctly classified (Figure 1C5). This level of failure is inevitable with a normal distribution of the background intensities. Accordingly, using three instead of two SDs results in the correct designation of almost the entire background population of pixels (data not shown).

We also tested all of the methods available in Fiji under Auto Local Threshold and found that the Phansalkar method [7] using the default settings to be the best (Figure 1C6), outperforming all others including the Bernsen, Mid Grey, Sauvola [8] and Niblack [9] methods (data not shown). The Phansalkar method correctly identified all the objects and only misclassified around 15% of the background pixels, mostly single pixels. The false colour scale and the graphs show that the Phansalkar method produces a threshold in the background regions that is higher than all other methods shown except the LMB+LB.SDx2 and the LM+SDx2. The latter however was set so high that the foreground was largely misclassified. Over foreground regions the Phansalkar method by contrast generates a lower threshold (Figure 1B4) than all but the LMB+LB.SDx2.

The success of the segmentation methods varies in different areas of the test image (Figure 1C), which is summarized in Table 1. This considers objects and background separately and for each also considers an annulus around the edge of the objects and the remaining background outside the annulus (Figure 2). The superiority of the Phansalkar and LMB plus twice the standard deviation of the local background is clear.

When performance is related to the size of objects it is noteworthy that the LM+SDx2 has a very sharp cut off (Figure 1E) only selecting objects appreciably smaller than the calculation radius. The local median and the LM are also less effective with larger objects, principally due to a failure to correctly segment their centres. Whereas the other three methods; the Phansalkar, LMB, LMB+LB.SDx2, are unaffected by the size of the objects.

### The LMT and its effect on colocalization measurements

Classifying pixels into foreground or background is an important step in measuring colocalization, Misclassification of pixels clearly influences any quantitation. In addition to being used to simply differentiate between foreground and background, the LMT has been used to set pixels below the threshold to zero [5]. This creates combinations of intensities not present in the original image (Figure 1D). The scattergrams show that the two original groupings of foreground (a1) and background (b1) then expand to seven, mostly at the expense of original background, which loses three quarters of its area (b2) with a reduction in the number of pixels present in its residual upper right quadrant. However only the combinations forming the background population (e) are correctly designated. The LMT also affects the original foreground (a1), with populations (c) and (g) breaking off after being incorrectly designated.

There are consequences for colocalization measurements when pixels are misclassified. The *r* of the original foreground (*a1*) is 0.000 but rises to 0.994 after applying the LMT, because (*a2*) and (*b2*) are now designated as foreground. The *H_coef_* is also affected, with the foreground measurement changing from 1.000 to 1.380 and when all pixels are included the change is from 1.813 to 3.297.

### Empty voxels and their effect when included in correlation measurements

**Figure 3:**
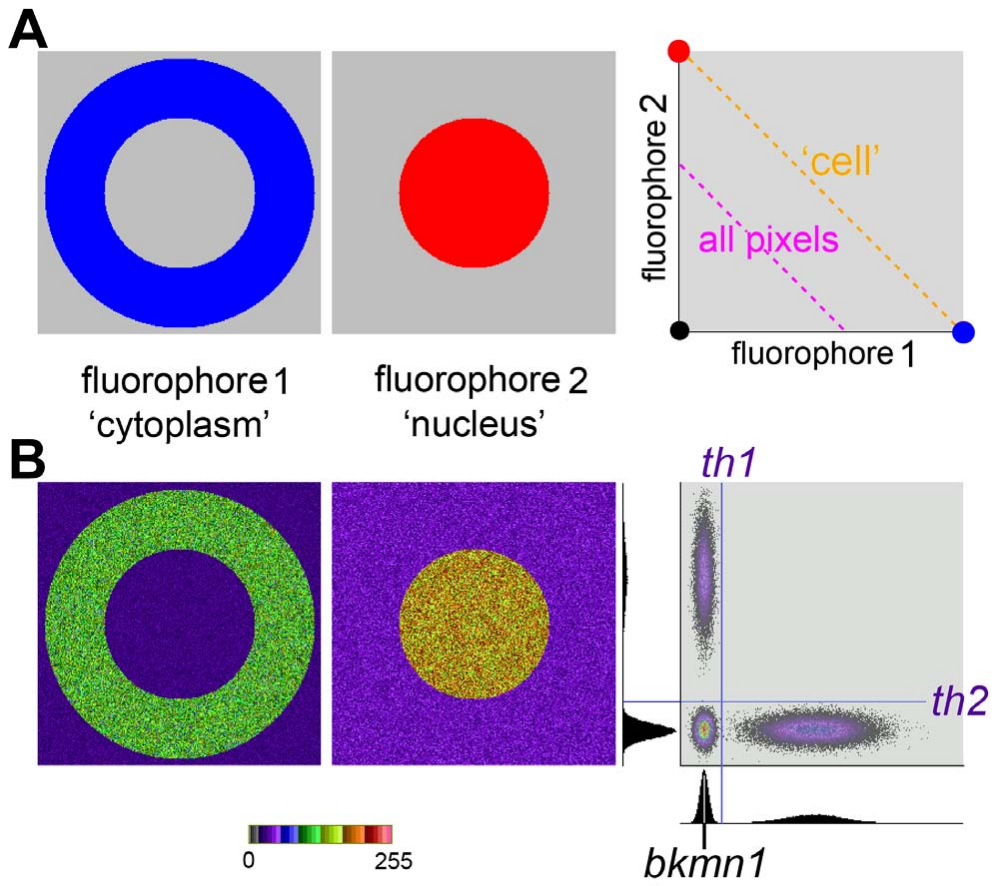
Correlation and empty voxels. (A). A cartoon cell with one ‘fluorophore’ (blue) in the ‘cytoplasm’, the second (red) in the ‘nucleus’ and no external fluorophore. The distribution of the intensities is shown in a scattergram with regression lines showing the linear best fit for the relationship within the ‘cell’ (‘nucleus’ and the ‘cytoplasm’) and a second line that also includes the pixels outside the ‘cell’. (B). Same pattern as in A but with a range of intensities in each region. Frequency distribution histograms for each fluorophore are aligned with the scattergram. Thresholds (*th*1 and *th2*) that separate the foreground and background were derived from the frequency distribution histograms for the two ‘fluorophores’. The background mean (*bkmn1*) for ‘fluorophore1’ is the lower intensity peak of the frequency distribution histogram.

To illustrate the detrimental effect of the inclusion of background pixels in correlation measurement a cartoon cell was created (Figure 3A): two fluorophores with uniform intensity in different compartments. The contents of the ‘nucleus’, ‘cytoplasm’ and exterior are uncorrelated. The ‘cell’, i.e. the ‘nucleus’ and the ‘cytoplasm’, has a perfect negative correlation (−1) with a co-occurrence of zero. The correlation falls to 0.483 when a third population, the pixels outside the cell, in which neither fluorophore is present, are included in the analysis. When the distribution of intensities within the ‘nucleus’ and ‘cytoplasm’ are not uniform *r* for the ‘cell’ falls from −1 to −0.892 (Figure 3B). Since the nominal fluorophores do not overlap the co-occurrence always is zero but different correlations can be measured. The images are supplied as Figure S1 and Figure S2.

**Figure 4:**
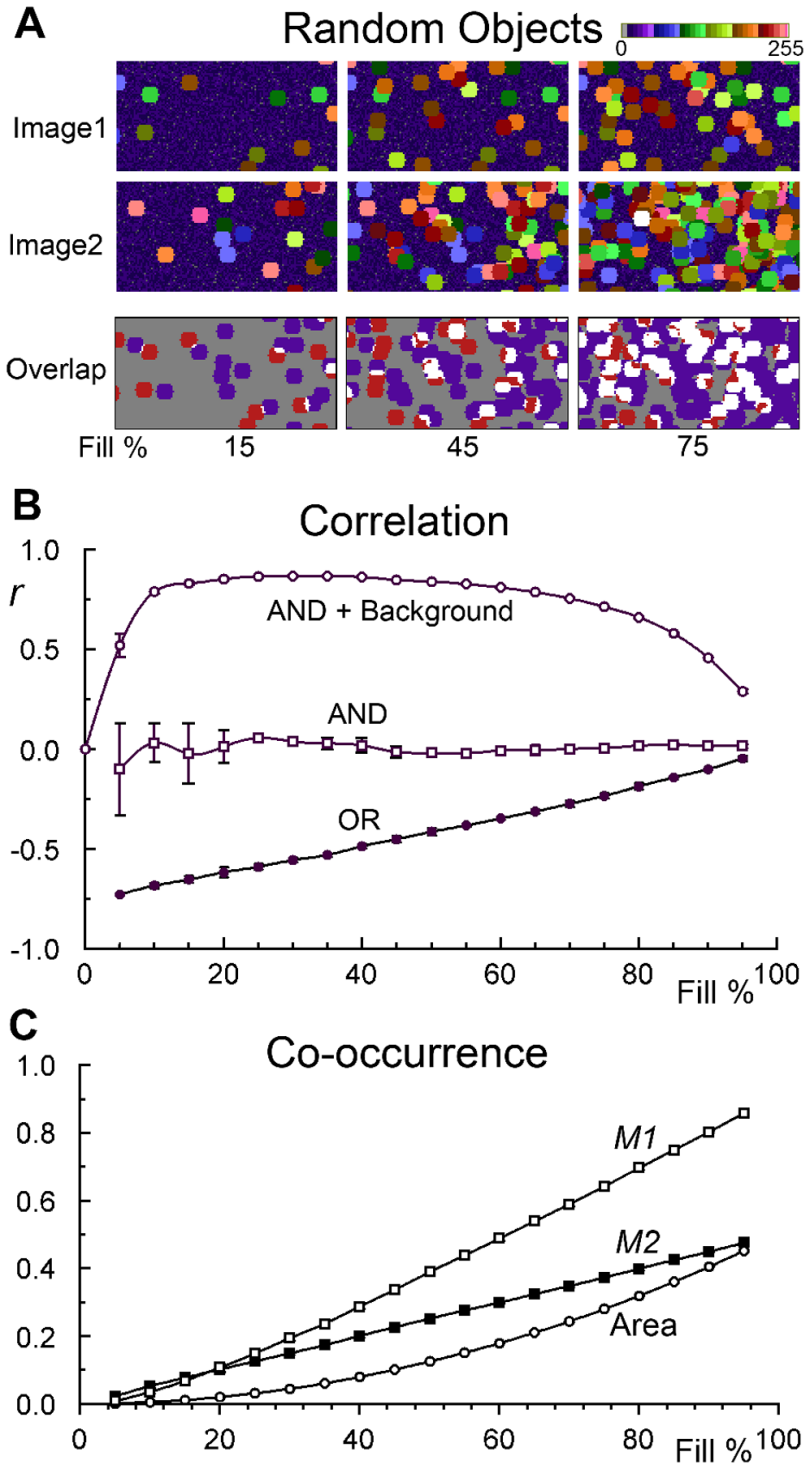
Which pixels to use in correlation measurements. (A) A series of uncorrelated paired images were created by inserting objects with a random intensity at random locations. The Fill% is the area occupied by objects in Image2, the corresponding occupancy of Image1 was half that of Image2. The panels show 128,64 pixel areas taken from the 512,512 originals. The two upper panels use false colour. The lowest panel shows objects from Image1 in red, objects from Image2 in blue and co-occurrence in white. (B) and (C) show correlation (*r*) and co-occurrence (*M1* and *M2*) for the image pairs illustrated in A. Measurements were made from three different areas: AND (co-occurrence), OR (either or both) and AND+ Background (AND plus pixels that are only background). The *r* for the background only pixels was always near zero and is omitted. Note the AND and OR cannot be measured with a Fill% of 0 and the Fill% of 100 is omitted - the expected value is zero. Data shown are means +SD, N = 4.

To address which pixels should be included in correlation analysis, a series of images were constructed by progressively inserting objects with random intensities at random locations in a pair of images (Figure 4A), distributions that are uncorrelated. The area covered is referred to as the Fill% which shows the occupancy in Image2, the Fill% of the associated Image1 was always half that of Image2. Measurements were made from the (i) AND areas (where both fluorophores are present), (ii) OR (one or both), (iii) AND plus background (Figure 4B).

The correlation between the pixels containing one or both fluorophores (OR) is initially strongly negative, but moves towards zero as the Fill% increases and the proportion of single fluorophore pixels falls. The AND pixels, those showing co-occurrence, remain uncorrelated regardless of the Fill%. The initially large SD in the AND measurements reflects the small number of pixels meeting the AND criteria:.001% at Fill% of 5,.005% at Fill% of 10,.011% at Fill% of 15 and finally reaching 45% with a Fill% of 95. The largest Fill% was 95 because completely filling an image by inserting random objects is impractical.

Combining the AND with the background only pixels produces a positive correlation that rises sharply and then slowly drops towards zero, reflecting the disappearance of background pixels when the images are filled with objects. The background pixels measured alone remain uncorrelated, as would be expected (data not shown).

Co-occurrence is reported in Figure 4C. *M1*, *M2* and the *Area* all rise progressively as the Fill% increases. The change in the co-occurrence *Area* is non linear, as it is the product of the Fill% for the two images i.e. if the Fill% of each image is 10 then the area of co-occurrence is 1%.

### A comparison of the *H_coef_* and r

**Figure 5:**
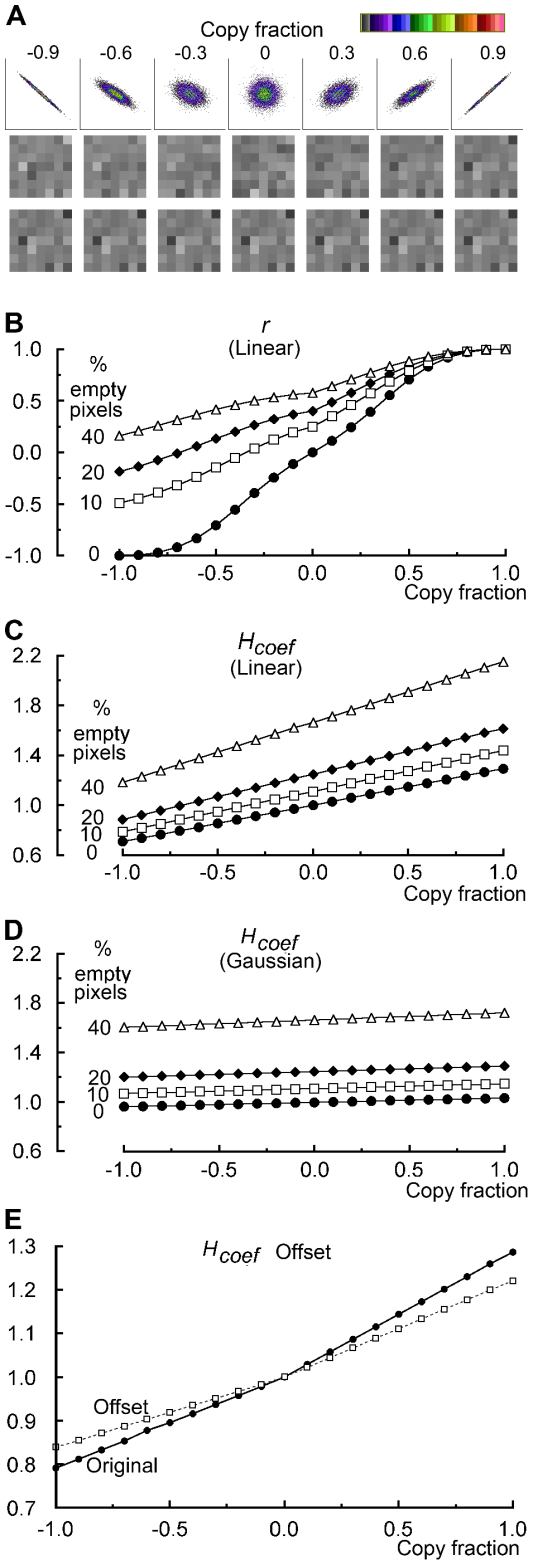
The effect of void pixels on the *H_coef_* and *r*. (A) The copy fraction was used to vary the correlation, shown using scattergrams. The range shown by the false colour scale is 0–124. The two lower panels show individual pixels from a small part of the corresponding images. Note that the lowest panel does not change. For more details see the Methods section. (B), (C) and (D) illustrate the effect of changing the copy fraction and in addition setting differing fractions of the pixels in both images to zero intensity. (A), (B) and (C) have linear distributions of intensities, with mean of 128 and a width of 200 while D has a mean of 128 with a Gaussian distribution with a SD of 24. (E) The effect of applying an offset of 32 to one image. Both images originally had a mean of 128, a linear distribution of intensities with a width of 200 and no empty pixels. *r* is unaffected by offset and the corresponding graph is the 0% in panel B.

The performance of *r* and the newly introduced *H_coef_* was compared (Figure 5) using a sequence of incrementally changing distributions (Figure 5A) produced by varying the copy fraction. When the original intensity distributions have a linear distributions (Figure 5B and C), the Pearson correlation coefficient *r* changes over its full range, −1 to +1, while the *H_coef_* has a more limited response, from 0.7 to 1.2, a smaller part of its full range which can extend from 0, no co-occurrence, towards infinity, when all the fluorescence co-occurs in a single voxel of a large image. The response detected by the *H_coef_* is even more limited when the distribution of intensities used in the simulated images is Gaussian, arguably more representative of biological images, rather than linear (Figure 5D). The reported change over the full correlation range is then only from 0.965 to 1.035. *r* with a Gaussian distribution is not shown since the difference from 5B is marginal.

Another notable difference is the effect of an offset, *r* is unaffected but the *H_coef_* is increased when the correlation is below 1 and reduced when the correlation is higher than 1 (Figure 5E).

The inclusion of empty voxels increases *r* most dramatically when the correlation is highly negative, but this shift progressively drops as the original correlation becomes more positive. The *H_coef_* responds quite differently with an almost identical shift over the full range of the copy fraction.

Overall the *H_coef_* responds to an offset, detects empty voxels and to some extent reports correlation. This multiplicity of responses means that different distributions can produce an *H_coef_* of 1.0 (Figure 5B and C), which nominally indicates a random distribution. Since dissimilar distributions produce the same value for the *H_coef_*, it is a poor descriptor of colocalization.

## Discussion

### Segmentation by thresholding

Setting intensities for thresholding is one of the hardest steps in image analysis. To test locally derived thresholds we created a simple image, circular objects with an appreciably higher intensity than the background, plus a dash of noise. Methods unable to segment such a simple images are unlikely to work with more demanding images and the simplicity of the test image allows the identification of the origin of failure or success.

As we predicted the LMT performed poorly, incorrectly designating around half the background pixels and many pixels within objects. The LMT really operates as a thick edge detection tool, efficiently designating background pixels in an annulus outside and inside objects but becomes less successful as the size of objects increases. More surprising was the poor performance of the LMT+SDx2 [10], [11], an increase expected to produce a threshold above most the background. Almost every background pixel is indeed correctly designated but the higher threshold then rejects all but the smallest objects. The edges are lost because when the calculation area for the local mean includes both background and foreground intensities the local SD becomes much larger than that of areas that are just foreground or background. And the object centres are also lost because raising the threshold by twice the relatively modest local SD of these fairly homogenous areas raises the threshold above the intensity of the objects. The result is that only objects with an area less than one sixth of that used to calculate the threshold are retained. This is similar to the local median filtering where a calculation area of at least five times the object size is recommended [12]. The two LM based and the local median methods cannot be recommended.

The most successful thresholding method is LMB+LB.SDx2. However this method appears to be self referential, requiring that background has been identified in order to segment the background. But the intensity range of the background pixels can be obtained from the local distribution of intensities in the calculation area; if the background forms a distinct low intensity peak, the peak can be taken as the local mean background and the local background SD comes from the width of the peak. Problems obviously arise, as they do for most local operators, when the local area is predominately foreground or background and there is only one intensity peak. This can be handled by either expanding the calculation radius until two peaks can be identified or by interpolating a threshold value from adjacent areas where the background peak can be identified. This is not a simple mechanistic method like the LMT and the algorithm is under development.

The Phansalkar method is very successful in separating background and foreground. It employs a complex combination of the local mean, local standard deviation and normalization [7]. The Phansalkar method is a development of the Sauvola method [8] which is in turn a variant of Niblack's method [9]. It cleverly sets a threshold that is below the local mean, to select the foreground but rejects the background by boosting the threshold at lower intensities. There is also scope for fine-tuning two of the parameters used in the Phansalkar method calculation [7].

In colocalization two thresholds may be needed, one to differentiate between the fluorophore's emissions and background, and a second to remove any intensity offset introduced by the background and/or detectors. Correlation is the simpler, requiring only the differentiation of foreground and background (*th1* and *t h2* in Figure 3B), since offsets do not affect the calculated value of *r* [13] when the calculation is performed correctly, i.e. includes only pixels in which both fluorophores are found. In Figure 3B there is some flexibility in exactly where this threshold (*th1* and *t h2*) is set with the distribution of intensities shown. However measures of co-occurrence like *M1* and *M2* require both thresholding to identify which intensities show the presence of fluorophores and a correction for any offset, since an uncorrected offset will alter co-occurrence measurements. Intensities below the threshold (*th1* and *t h2*) should be set to zero then any offset (*bkmn1* and *bkmn2*, the second is not shown) corresponding to the background means should be subtracted from all the pixels above the threshold. Subtracting the background mean from every pixel would merge populations (*b2*), (*d*), (*e*) and (*f*) in Figure 1D. If the background is uniform the background mean (*bkmn1* in Figure 3) should simply be subtracted [14] but biology and images may be unobliging [10], and require a localized calculation of the background mean.

### Correlation and co-occurrence

The case that colocalization should be split into co-occurrence and correlation is strengthened by the demonstration that, with objects inserted at random locations in the individual images, the correlation, measured from the pixels that show co-occurrence, correctly remains constant at around zero while the co-occurrence progressively increases. This supports our assertion made in the introduction that zero correlation is compatible with substantial co-occurrence.

It is also important to note that with these simulated images the correct correlation, which is known a priori from the mechanism used to create the images, was only reported correctly when r was derived from the AND pixels, i.e. those containing both fluorophores.

### Empty voxels and correlation measurements

A common view is that empty voxels, those without either fluorophore, should be included in measurements [15], expressed for instance as ”And a negative PCC value indicates that the distributions of the two probes are inversely related, … for example, if one protein is restricted to the cell nucleus, and a second is localized in the cell cytosol” [16]. This is illustrated in Figure 3 by a cartoon cell with one fluorophore in the nucleus and a second in the cytoplasm and a scattergram showing the distribution of paired intensities across the whole cell.

The opposing view is that considering correlation under these circumstances conveys no useful information, given that the nominal fluorophores are in different cellular compartments and cannot interact [17], [18]. The scattergrams on the right of Figure 5A and Figure 3 both have strong negative correlations, the first has a continuous distribution but in the second the two nominal fluorophores never co-occur.

### Reasons for dismissing negative correlations in the absence of co-occurrence

Early work with correlation found a positive relationship between the length of arms and legs [19]. Choosing subjects with arms but no legs and legs but no arms would produce a completely different result, a negative correlation. A result that clearly has no relevance to normal subjects. Further, if the intensity distribution of two fluorophores each isolated within separate compartments changes then we show that the measured correlation also changes. So if the first measurement describes some interaction of the fluorophores it follows that the second measurement must indicate a change in this interaction. But there cannot actually be a change in any type of interaction since the fluorophores are not in the same place. Reporting a negative correlation in the absence of co-occurrence is misleading because it suggests the magnitude is meaningful, but there are no degrees of not being in the same place in pixel-based calculations. Interactions at a distance would require different measurements. However, real negative correlations do exist and should not be confused with meaningless negative correlations. Accordingly we strongly suggest that correlations should only be reported when there is co-occurrence. When there is no co-occurrence, reporting the absence of co-occurrence is a full and sufficient description.

### What happens when there is not a continuous distribution of intensities?

Mathematically *r* considers how the intensities vary around their respective mean. The expectation is that each fluorophore has a continuous distribution of intensities, ideally a bivariate normal distribution. *r* is not robust in the sense that outliers, values distant from the mean, have a strong influence on the overall measurement [18], [20]. These outliers explain why the correlation measured from the distribution shown in Figure 3A and B is meaningless, relative to the mean the scattergram shows only outliers.

*r* only considers a linear relationship, shown the by the regression line ‘*cell*’ in Figure 3. But the line of best fit joins two disconnected distributions which when examined individually are uncorrelated. While the second regression line ‘*all*’ (*r −0.483*) does not even approach any of the data points. This is an example of Simpson's paradox in which a trend present in individual populations disappears or reverses when the populations are merged [21]. The use of *r* is problematic when more than one relationship is present [13]. For instance two strongly positive correlations when measured concurrently can have a much lower correlation and two uncorrelated populations, when treated as a single population, can have a strongly positive correlation. This provides another argument for disregarding the measurement derived from the distributions shown in Figure 3 - there are actually several relationships, or non-relationships, in play.

In summary, the magnitude of a correlation measured between two fluorophores that never appear in the same place conveys no more information than simply saying that they are not found together which can more accurately be described by their (lack of) co-occurrence. The Manders *M1* and *M2* coefficients, both unsurprisingly report zero co-occurrences for the distributions shown in Figure 3.

### Negative correlations are rarely the result of repulsion

Negative correlations are often ascribed to molecular repulsion or avoidance as are values of the *H_coef_* below 1 [5]. True repulsion requires that molecules in a homogenous volume avoid each other, a more dramatic separation than indifference. But molecules may appear in different biological compartments simply because access is restricted or they are delivered elsewhere, i.e. molecular repulsion need not be invoked. Biologically values of the *H_coef_* below 1 or negative correlations can originate from opposing concentration gradients of morphogens but these are not attributable to repulsion [22]. An enzyme transforming a substrate could also produce a negative correlation or an *H_coef_* below 1, independently of repulsion. Indeed if repulsion, as distinct from simply not interacting, does occur its operating scale would be such that images of cells with voxels with sides of tens of hundreds of nm would be unlikely to detect it. Even with single molecule localisation imaging it would be difficult to establish the existence of repulsion.

### The *H_coef_* and r

The inclusion of empty voxels increases *r* most dramatically when the correlation is highly negative, which is a strong argument for restricting *r* to voxels that show co-occurrence. The *H_coef_* responds to a combination of offset, empty voxels and correlation. This makes it unsuitable for comparing populations since the same *H_coef_* value can arise from appreciably different distributions. A random distribution is only one of many possibilities producing the value 1, so the *H_coef_* describes the underlying relationship of intensities poorly.

The *H_coef_*, unlike *r*, requires the inclusion of empty voxels. However, there are two categories of empty voxels; voxels that are empty because the molecules of interest are unable to reach them and voxels to which both molecules have access but still remain empty. For reasons outlined above, the second category is likely to be extremely rare in biology. This means that there is no practical case for using the *H_coef_*.

### The H_coef_ and the Manders overlap coefficient



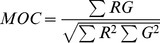
(2)The *H_coef_* (eq. 1) and the *MOC* have obvious mathematical similarities. The numerator of both includes the sum of the product of the intensities of the two fluorophores in every voxel. In both cases voxels lacking one or both fluorophores do not affect the numerator since their product is zero, and the numerator is maximized when high intensities coincide. For a fixed number of voxels and a constant amount of fluorophore the denominator will be constant and the measurements of the *H_coef_* or the *MOC* will follow the same trend and report on the combinations of intensities found across the population of voxels.

Important differences between the two coefficients is the presence of *N* in the numerator of the *H_coef_*, and changes to the denominator which means that the *H_coef_* increases if empty voxels are present while the *MOC* is unaffected.

An important similarity between the *MOC* and the *H_coef_* is that both require that the intensities held in each voxel are related to the number of photons emitted by the fluorophores. Therefore empty pixels should have an intensity of zero, so any offset produced by background must be removed before colocalization is calculated. An offset increases the *H_coef_* when the correlation is low but the *H_coef_* is decreased as the correlation becomes more positive (Figure 5). In contrast, an offset always makes the *MOC* more positive [13].

A criticism of the *MOC* recognized by Herce et al. and others is that it is hard to interpret [5], [13], [23]. This arises because it is responsive to both co-occurrence and correlation. The same applies to the *H_coef_.*

### What does the *H_coef_* measure?

The *H_coef_* was derived and discussed in terms of molecular interaction, either attraction or repulsion. In a homogenous medium, perhaps a solution in a test tube, the relative distribution of the two fluorophores might reflect molecular interaction. But biology is the antithesis of homogeneity and the composition of the small volume of a specimen that corresponds to a voxel is likely to be highly variable. Biology defeats the case for the *H_coef_*.

Let us first consider homogenous media: two different molecules with a very high mutual affinity will pair off and the number of pairs per voxel would be expected to show a Poisson distribution. This will produce a high but not the highest value for the *H_coef_*. Lower densities of molecules will produce a higher *H_coe_*_f_, since not all pixels will be occupied, although the molecular interaction is unaltered. The highest *H_coef_* will occur when all the paired molecules are concentrated in a single voxel. However such a concentration is unlikely to arise from the interaction between the two molecules, which is the focus of colocalization measurements, unless the paired molecules themselves interact to form clusters or polymers. So, identical molecular interactions could generate a range of *H_coef_* values in a homogenous medium.

The exclusion of both molecules from part of the volume analysed will clearly increase the *H_coef_*, but exclusion has nothing to do with molecular interactions in the remaining area.

An equal number of molecules with high mutual affinities will form pairs, which would be recognized by the *H_coef_* and might, correctly, be considered a molecular interaction. But if the numbers of each species are not equal then there will be spare molecules drifting around, presumably with their own Poisson distribution, and the *H_coef_* would fall, potentially misinterpretable as a change in molecular interaction.

Now let us consider a cell. The distribution of molecules in cells is not random. Every protein that is destined for an organelle other than the cytoplasm has in its sequence a targeting motif to ensure delivery to the appropriate cellular compartment [24]–[26]. Once delivered molecules are generally retained. Small punctate cytoplasmic objects observed in a confocal image might well show the presence of both fluorophores but to conclude that the distribution is due to molecular interactions between the fluorophores is naive – they may simply co-occur. Not because they interact but because they are delivered to and reside there or simply have similar physico-chemical properties. They may or may not interact but concluding that they interact because the *H_coef_* measured from the whole cell or the cytoplasmic compartment containing organelles shows a non-random distribution is a mistake. Even if the measurement is restricted to one type of organelle a positive *r* or a high *H_coef_* suggests, but does not definitively prove a molecular interaction.

Even when a biologically relevant ROI is used a false high *H_coef_* or *r* is likely, or at least needs to be excluded. The reason is prosaic; different amounts of an organelle may be present in each voxel. For instance, the plasma membrane has a thickness of around 5 nm and folding, protrusions and invaginations cause a substantial variation in the plasma membrane content of voxels, producing a positive correlation or a better than random *H_coef_* between two molecules randomly distributed in the plasma membrane [27].

### The *H_coef_* and images of biological origin

The *H_coef_*, has only been tested on one sequence of biological images, a single nucleus followed over part of the cell cycle, with one fluorophore marking sites of active replication and a second showing heterochromatin–rich regions [5]. The changes in distribution are clear, with the brief appearance of small punctate patches containing both fluorophores in late S phase. Two local thresholds were applied by Herce et al, the LMT and LMT×1.2, with the declared intention of reducing background noise by setting pixels below the threshold to zero, presumably because their intensity was considered to be due to background. Note that voxels with zero intensity affect the *H_coe_*_f_. But setting pixels below the threshold to zero creates an anomaly: voxels with a slightly higher intensity retain their original intensity including any offset/background. As we have shown the LMT is a very poor method of thresholding, increasing it by 20% will improve the rejection of background but lacks a clear rationale and may compromise parts of the foreground. The LMT identifies small punctate objects, but one of the fluorophores had a diffuse distribution, which leads to many foreground voxels being misclassified as background voxels, with unknown effects on colocalization measurements.

Using the LMTx1.2 the *H_coef_* reported an appreciable change in colocalization in late S phase, with *r* reporting a qualitatively similar change. However when using the unenhanced LMT the change previously recorded by the *H_coef_* disappeared, although it was still shown by *r*, though the pattern of correlation during the cell cycle was altered. Nonetheless Herce et al. concluded that the *H*_coef_ performed better than *r*. The results with *r* are also questionable since they use the same two variants of the LMT and then included all the voxels in the measurements. Taking into account the inappropriate use of the LMT, problems with the LMT, failure to correct for offset and problems with the application of *r*, we suggest that the quantification reported by Herce et al. with the *H_coef_* and *r* are questionable. Given the very obvious changes in co-occurrence, the Manders *M1* and *M2* pair might have provided a clearer description of the changes during the cell cycle.

## Conclusions

Thresholding to differentiate between foreground and background is a critical step in quantifying colocalization. The LMT can misclassify about half the background pixels and a similar fraction at the centre of larger objects. The background is correctly classified when the threshold is increased by adding twice the local standard deviation to the LMT, but at the expense of excluding all but the smallest foreground objects. The best local thresholding methods were the Phansalkar and the local mean background plus twice the local background standard deviation.

The newly launched *H_coef_* like the *MOC*, *M1* and *M2* is sensitive to offsets and therefore requires both thresholding and the removal of the mean background intensity to correct for any offset. The offset can be obtained from the lowest intensity peak of a frequency distribution histogram.

Correlation measurements only correctly report the relationship between the fluorophores from the pixels in which the two fluorophores co-occur. Including empty pixels and pixels with only one fluorophore misrepresents the underlying relationship between the fluorophores.

Molecular interaction is only one of the possible explanations for positive correlations and *H_coef_* values above 1. Inhomogeneity and molecular targeting strongly influence cellular distributions but molecular repulsion is likely to be insignificant.

The case for using the *H*_coef_ for colocalization partly depends on finding deficiencies in existing coefficients. However no superiority of the *H_coef_* over the *r or M1* and *M2* coefficients has been demonstrated and its value in the analysis of images of biological origin is unproven.

## Methods

### Intensity Thresholding

A simple test image (629,256 pixels, byte) (Figure 1A2) was created by combining circular foreground objects of incrementally increasing diameters (3 to 39 pixels) and a uniform intensity of 128 (Figure 1A1) with background noise (mean 32, Poisson noise based on 32 quanta, SD 5.67) and used to evaluate different local thresholding methods. Local thresholds (Figure 1B), calculated over a circle with a diameter of 21 pixels, were produced using Fiji [28] and applied by setting intensities below the threshold to zero, while leaving those above threshold unaltered (Figure 1C). The thresholding was quantitated using Semper6p (Synoptics, Cambridge, UK) with six different regions of interest (ROIs) created from the original test image: the objects and background, with each divided into an annulus (inside or outside extending 10.5 pixels) around the edge of the objects (Figure 2) and the residual area (the centres of objects or the background minus the outer annulus).

Two copies of the test image with the noise component created separately for each copy were used for colocalization measurements. The images are supplied as Figure S3 and Figure S4.

### Random Objects

Uncorrelated distributions were generated by inserting circular objects (radius 5 pixels) at random locations within 512,512 byte images. The intensities of each object were taken from a sequence of random numbers with a uniform distribution of intensities between 64 and 254.

Images had a background with a mean of 16 and a variability based on 16 quanta. The Pearson correlation coefficients (*r*) was used to follow the change in correlation in different ROIs as objects were progressively added to the images. The images are supplied as Figure S5, Figure S6, Figure S7, Figure S8, Figure S9, Figure S10, Figure S11 and Figure S12. Panel A in Figure 4 shows a small area from the sequence of images used in B and C.

The supplementary figures contain the whole images of the full sequence of images.

### Test images with varying correlation

A range of paired images with correlations that incrementally vary from −1 to 1 (Figure 5) were produced from two independently generated distributions by replacing differing fractions (the copy fraction, *cf*) of the difference from the mean in each pixel of one image with the same fraction of the difference from the mean of the corresponding pixel of the other image:
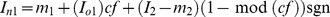
(3)


*m_1_* and *m_2_* are the mean intensity of image_1_ and image_2,_

*cf* is the copy fraction with a range of −1 to +1,

*I_n1_* and *I_o1_* are the new and original intensities in individual pixels in the first image and *I_2_* the intensity in the second image.

*mod* is the modulus.

*sgn* is either +1 or −1, creating positive or negative correlations.

Provision was also made for setting the intensities of a fraction of the pixels in both images to zero and for offsetting the intensities of one image by a constant.

Images with a linear distribution had a uniform distribution of intensities from 0–247. Images with a Gaussian distribution had a distribution of 23–233 with a SD of 24.

The images for the Gaussian and linear distributions are supplied as Figure S13, Figure S14, Figure S15 and Figure S16.

### Image analysis

Colocalization measurements were made using the *H_coef_*, *r, M1 and M2* with software based on a Semper6p kernel (Synoptics, Cambridge, UK) and the ImageJ plugin Auto Local Threshold (Gabriel Landini et al.). Image processing and analysis was implemented using Semper6p kernel or ImageJ [29]. File S1 holds a program that runs within the Semper6p image analysis software and creates the Images used in Figure 4 and File S2 a program that creates the images used for Figure 5. The Semper protocols for the image analysis can be found as File S3 and File S4.

## Supporting Information

Figure S1 **Image of the nucleus.** The nucleus image from Figure 3B. In greyscale, rather than false colour.
(TIF)

Figure S2 **Image of the cytoplasm.** The cytoplasm image from Figure 3B. In greyscale, not false colour.
(TIF)

Figure S3 **Small image of objects and background.** Objects and background from Figure 1A.
(TIF)

Figure S4 **Large image of objects and background.** The wider range of sizes and eight sets of each size used to make the graph shown in Figure 1E.
(TIF)

Figure S5 **Image one of the sequence of the incremental increase in Fill% set a.** The whole image of the full sequence of images covering the incremental increase in the Fill% shown in Figure 4A. The image is a greyscale tif stack with the lowest Fill% in the first image, each subsequent image increases the Fill% by 5. Image 1, set a.
(TIF)

Figure S6 **Image one of the sequence of the incremental increase in Fill% set b.** The whole image of the full sequence of images covering the incremental increase in the Fill% shown in Figure 4A. The image is a greyscale tif stack with the lowest Fill% in the first image, each subsequent image increases the Fill% by 5. Image 1, set b.
(TIF)

Figure S7 **Image one of the sequence of the incremental increase in Fill% set c.** The whole image of the full sequence of images covering the incremental increase in the Fill% shown in Figure 4A. The image is a greyscale tif stack with the lowest Fill% in the first image, each subsequent image increases the Fill% by 5. Image 1, set c.
(TIF)

Figure S8 **Image one of the sequence of the incremental increase in Fill% set d.** The whole image of the full sequence of images covering the incremental increase in the Fill% shown in Figure 4A. The image is a greyscale tif stack with the lowest Fill% in the first image, each subsequent image increases the Fill% by 5. Image 1, set d.
(TIF)

Figure S9 **Image two of the sequence of the incremental increase in Fill% set a.** The whole image of the full sequence of images covering the incremental increase in the Fill% shown in Figure 4A. The image is a greyscale tif stack with the lowest Fill% in the first image, each subsequent image increases the Fill% by 5. Image 2, set a.
(TIF)

Figure S10 **Image two of the sequence of the incremental increase in Fill% set b.** The whole image of the full sequence of images covering the incremental increase in the Fill% shown in Figure 4A. The image is a greyscale tif stack with the lowest Fill% in the first image, each subsequent image increases the Fill% by 5. Image 2, set b.
(TIF)

Figure S11 **Image two of the sequence of the incremental increase in Fill% set c.** The whole image of the full sequence of images covering the incremental increase in the Fill% shown in Figure 4A. The image is a greyscale tif stack with the lowest Fill% in the first image, each subsequent image increases the Fill% by 5. Image 2, set c.
(TIF)

Figure S12 **Image two of the sequence of the incremental increase in Fill% set d.** The whole image of the full sequence of images covering the incremental increase in the Fill% shown in Figure 4A. The image is a greyscale tif stack with the lowest Fill% in the first image, each subsequent image increases the Fill% by 5. Image 2, set d.
(TIF)

Figure S13 **Red image Gaussian distribution.** The single single red image of the Gaussian distribution in Figure 5.
(TIF)

Figure S14 **Stack of green images Gaussian distribution.** The stack of 21 green images with incremental steps of 0.1, the first with a copy fraction of 1.0 and the last with a copy fraction of −1.0 of the Gaussian distribution in Figure 5.
(TIF)

Figure S15 **Red image linear distribution.** The single single red image of the linear distribution in Figure 5.
(TIF)

Figure S16 **Stack of green images linear distribution.** The stack of 21 green images with incremental steps of 0.1, the first with a copy fraction of 1.0 and the last with a copy fraction of −1.0 of the linear distribution in Figure 5.
(TIF)

File S1 **Coloclization analysis program.** The file holds the text for a program that calculates the colocalization coefficients within Sepmper6p.
(TXT)

File S2 **Memory location program.** The file holds a very short program that is called by File S1 that finds a free memory location for an image or data file.
(TXT)

File S3 **Random blob image generating program.** The file holds a program that runs within the Semper6p image analysis software and creates the images used in Figure 4.
(TXT)

File S4 **Copy fraction image generating program.** The file holds a program that runs within the Semper6p image analysis software and creates the images used in Figure 5.
(TXT)
