# Supplementary figures and images for: Quantifying Colocalization: Thresholding, Void Voxels and the Hcoef
Source: PLoS One. 2014 Nov 6;9(11):e111983. doi: 10.1371/journal.pone.0111983 (PMC4222960; doi:10.1371/journal.pone.0111983)

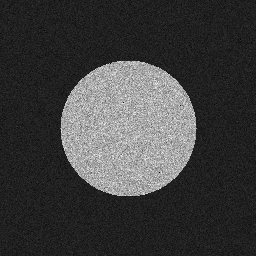

Supplement: Figure S1 — Image of the nucleus. The nucleus image from Figure 3B. In greyscale, rather than false colour. (TIF) [file pone.0111983.s001.tif]

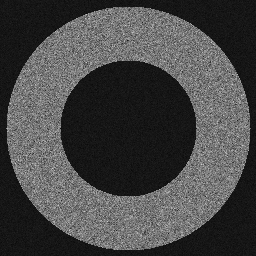

Supplement: Figure S2 — Image of the cytoplasm. The cytoplasm image from Figure 3B. In greyscale, not false colour. (TIF) [file pone.0111983.s002.tif]

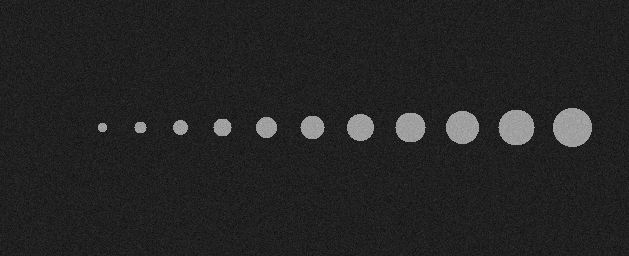

Supplement: Figure S3 — Small image of objects and background. Objects and background from Figure 1A. (TIF) [file pone.0111983.s003.tif]

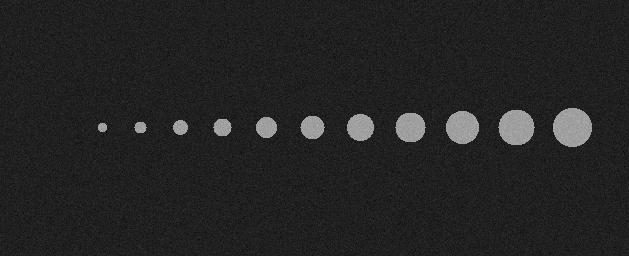

Supplement: Figure S4 — Large image of objects and background. The wider range of sizes and eight sets of each size used to make the graph shown in Figure 1E. (TIF) [file pone.0111983.s004.tif]

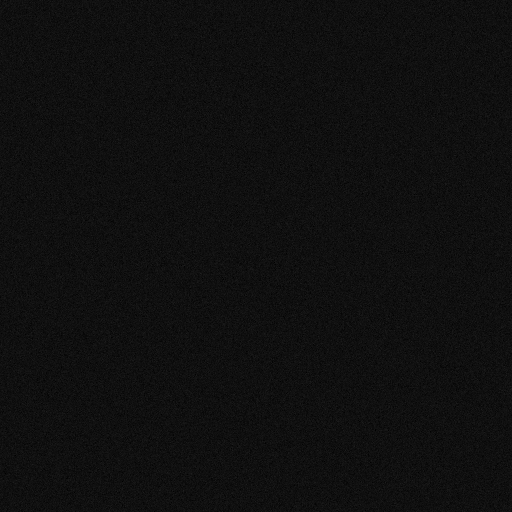

Supplement: Figure S5 — Image one of the sequence of the incremental increase in Fill% set a. The whole image of the full sequence of images covering the incremental increase in the Fill% shown in Figure 4A. The image is a greyscale tif stack with the lowest Fill% in the first image, each subsequent image increases the Fill% by 5. Image 1, set a. (TIF) [file pone.0111983.s005.tif]

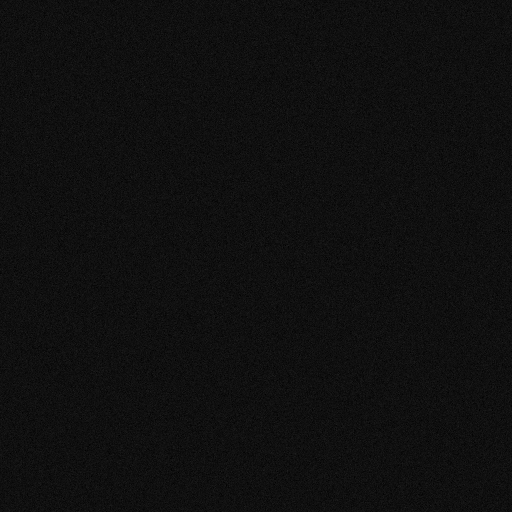

Supplement: Figure S6 — Image one of the sequence of the incremental increase in Fill% set b. The whole image of the full sequence of images covering the incremental increase in the Fill% shown in Figure 4A. The image is a greyscale tif stack with the lowest Fill% in the first image, each subsequent image increases the Fill% by 5. Image 1, set b. (TIF) [file pone.0111983.s006.tif]

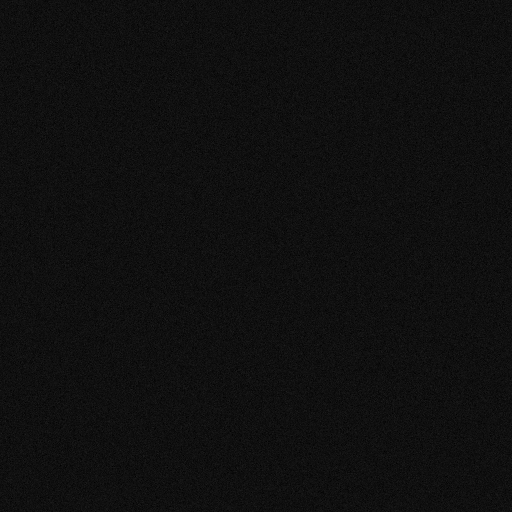

Supplement: Figure S7 — Image one of the sequence of the incremental increase in Fill% set c. The whole image of the full sequence of images covering the incremental increase in the Fill% shown in Figure 4A. The image is a greyscale tif stack with the lowest Fill% in the first image, each subsequent image increases the Fill% by 5. Image 1, set c. (TIF) [file pone.0111983.s007.tif]

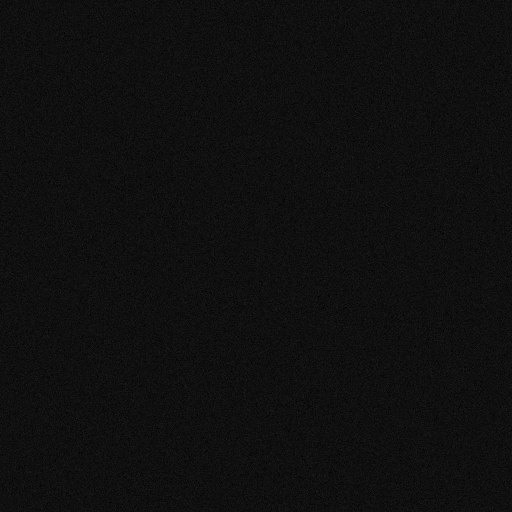

Supplement: Figure S8 — Image one of the sequence of the incremental increase in Fill% set d. The whole image of the full sequence of images covering the incremental increase in the Fill% shown in Figure 4A. The image is a greyscale tif stack with the lowest Fill% in the first image, each subsequent image increases the Fill% by 5. Image 1, set d. (TIF) [file pone.0111983.s008.tif]

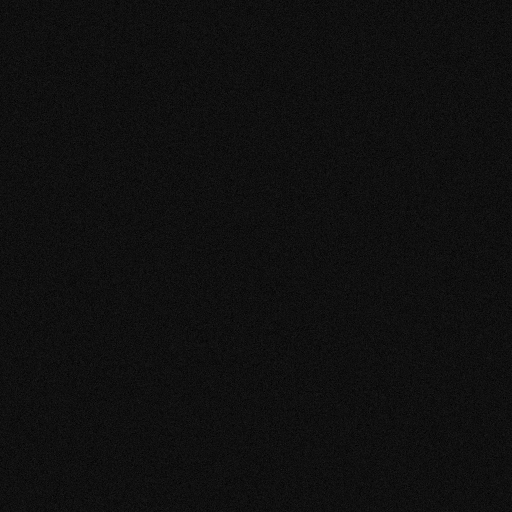

Supplement: Figure S9 — Image two of the sequence of the incremental increase in Fill% set a. The whole image of the full sequence of images covering the incremental increase in the Fill% shown in Figure 4A. The image is a greyscale tif stack with the lowest Fill% in the first image, each subsequent image increases the Fill% by 5. Image 2, set a. (TIF) [file pone.0111983.s009.tif]

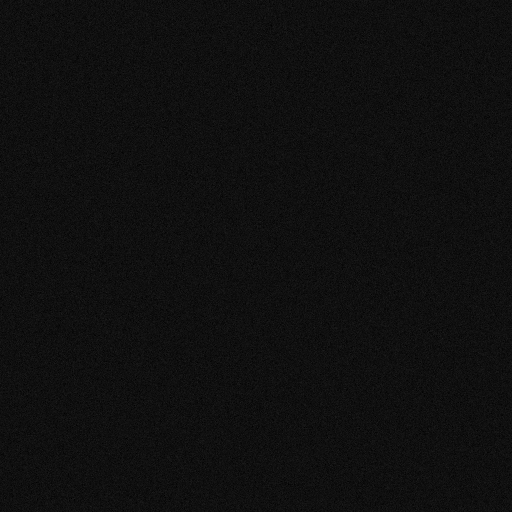

Supplement: Figure S10 — Image two of the sequence of the incremental increase in Fill% set b. The whole image of the full sequence of images covering the incremental increase in the Fill% shown in Figure 4A. The image is a greyscale tif stack with the lowest Fill% in the first image, each subsequent image increases the Fill% by 5. Image 2, set b. (TIF) [file pone.0111983.s010.tif]

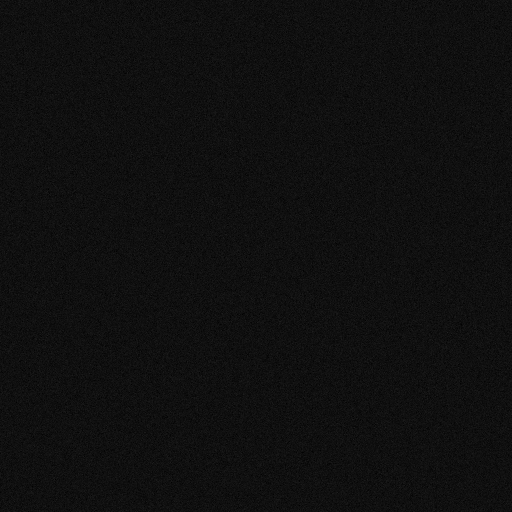

Supplement: Figure S11 — Image two of the sequence of the incremental increase in Fill% set c. The whole image of the full sequence of images covering the incremental increase in the Fill% shown in Figure 4A. The image is a greyscale tif stack with the lowest Fill% in the first image, each subsequent image increases the Fill% by 5. Image 2, set c. (TIF) [file pone.0111983.s011.tif]

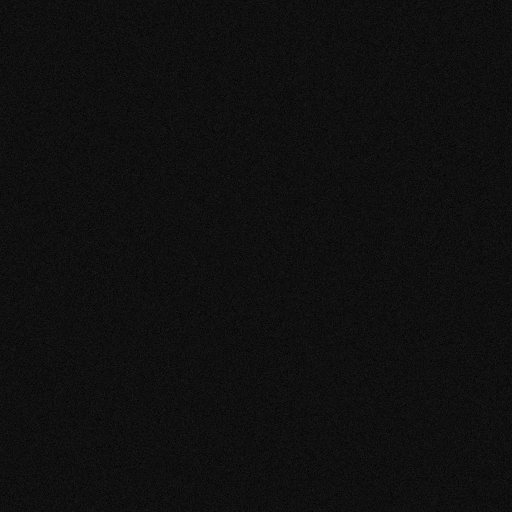

Supplement: Figure S12 — Image two of the sequence of the incremental increase in Fill% set d. The whole image of the full sequence of images covering the incremental increase in the Fill% shown in Figure 4A. The image is a greyscale tif stack with the lowest Fill% in the first image, each subsequent image increases the Fill% by 5. Image 2, set d. (TIF) [file pone.0111983.s012.tif]

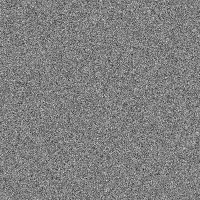

Supplement: Figure S13 — Red image Gaussian distribution. The single single red image of the Gaussian distribution in Figure 5. (TIF) [file pone.0111983.s013.tif]

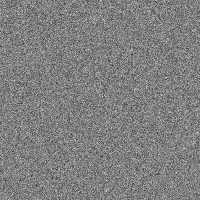

Supplement: Figure S14 — Stack of green images Gaussian distribution. The stack of 21 green images with incremental steps of 0.1, the first with a copy fraction of 1.0 and the last with a copy fraction of −1.0 of the Gaussian distribution in Figure 5. (TIF) [file pone.0111983.s014.tif]

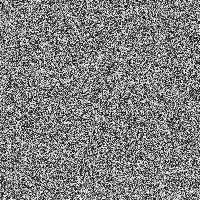

Supplement: Figure S15 — Red image linear distribution. The single single red image of the linear distribution in Figure 5. (TIF) [file pone.0111983.s015.tif]

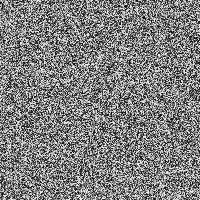

Supplement: Figure S16 — Stack of green images linear distribution. The stack of 21 green images with incremental steps of 0.1, the first with a copy fraction of 1.0 and the last with a copy fraction of −1.0 of the linear distribution in Figure 5. (TIF) [file pone.0111983.s016.tif]
